# Gene Expression of the Tumour Suppressor LKB1 Is Mediated by Sp1, NF-Y and FOXO Transcription Factors

**DOI:** 10.1371/journal.pone.0032590

**Published:** 2012-03-06

**Authors:** Nicolas Lützner, Johanna De-Castro Arce, Frank Rösl

**Affiliations:** Research Program Infections and Cancer, German Cancer Research Center (DKFZ), Heidelberg, Germany; Memorial Sloan Kettering Cancer Center, United States of America

## Abstract

The serine/threonine kinase LKB1 is a tumour suppressor that regulates multiple biological pathways, including cell cycle control, cell polarity and energy metabolism by direct phosphorylation of 14 different AMP-activated protein kinase (AMPK) family members. Although many downstream targets have been described, the regulation of LKB1 gene expression is still poorly understood. In this study, we performed a functional analysis of the human LKB1 upstream regulatory region. We used 200 base pair deletion constructs of the 5′-flanking region fused to a luciferase reporter to identify the core promoter. It encompasses nucleotides −345 to +52 relative to the transcription start site and coincides with a DNase I hypersensitive site. Based on extensive deletion and substitution mutant analysis of the LKB1 promoter, we identified four *cis*-acting elements which are critical for transcriptional activation. Using electrophoretic mobility shift assays as well as chromatin immunoprecipitations, we demonstrate that the transcription factors Sp1, NF-Y and two forkhead box O (FOXO) family members FOXO3 and FOXO4 bind to these elements. Overexpression of these factors significantly increased the LKB1 promoter activity. Conversely, small interfering RNAs directed against NF-Y alpha and the two FOXO proteins greatly reduced endogenous LKB1 expression and phosphorylation of LKB1's main substrate AMPK in three different cell lines. Taken together, these results demonstrate that Sp1, NF-Y and FOXO transcription factors are involved in the regulation of LKB1 transcription.

## Introduction

Liver Kinase B1 (LKB1, also called STK11) was initially identified as the tumour suppressor gene mutated in the inherited Peutz-Jeghers cancer syndrome, an autosomal dominant genetic disorder [1], [2], [3], [4]. It encodes a ubiquitously expressed and evolutionary well conserved protein kinase that is also inactivated in a large percentage of sporadic lung and cervical carcinomas [5], [6], [7], [8]. Indeed, examining the HPV18-positive cervical carcinoma cell line HeLa, LKB1 is not transcribed. Transcription, however, can be reconstituted by *trans* complementation [9] after somatic cell hybridization with normal human fibroblasts, leading to cellular hybrids with a non-tumourigenic phenotype. Tumourigenic segregants derived from the same hybrids again have lost LKB1 expression, suggesting that LKB1 down-regulation may favor progression towards malignancy [6].

Furthermore, LKB1 (+/−) mice develop hepatocellular carcinomas after loss of heterozygosity and loss of LKB1 also correlates with increased metastasis in a well-studied mouse model of lung carcinogenesis [10]. In this model, LKB1-deficient tumours showed even more frequent metastasis than tumours lacking the tumour suppressor p53 [11]. Known mechanisms that explain how LKB1 operates as a tumour suppressor, mainly depend on direct phosphorylation of different AMP-activated protein kinase (AMPK) family members [12], [13], [14], [15]. AMPK is a multi-component enzyme complex that acts as metabolic stress-sensor. Once activated, AMPK switches off many ATP-utilizing processes in order to sustain energy homeostasis. AMP binding allosterically activates AMPK, facilitating the binding of upstream kinases that enhance its activity [16].

Although various downstream targets such as the mammalian target-of-rapamycin (mTOR) pathway have been studied in detail [14], the regulation of LKB1 gene expression is still poorly understood. Hence, analysis of the transcriptional regulation of LKB1 should not only be helpful to identify important *trans*-acting regulatory proteins that can alter gene expression, but also to define critical *cis*-regulatory regions indispensable for its transcriptional control. Such regions could be affected in a variety of cases in which LKB1 was inactivated without having mutations within the coding sequence [17], [18]. Here, gene silencing via *de novo* DNA-methylation of CpG-rich stretches could be such a scenario [19]. Therefore, identification and characterization of the LKB1 promoter and transcriptional regulators is not only important to unravel the complexity of LKB1 gene silencing, but also to understand how upstream regulatory proteins mediate metabolic sensoring of nutritional depletion and in turn cell cycle control.

In this study we performed a functional analysis of the LKB1 promoter and identified distinct *cis*-regulatory elements, including three CCAAT boxes and a non-canonical GC-box that critically affected LKB1 gene expression. These elements bind NF-Y and Sp1, representing two ubiquitous transcription factors involved in the regulation of various genes [20], [21]. Furthermore, two forkhead box O (FOXO) transcription factors that bind the LKB1 promoter were identified. FOXO3 and FOXO4 activated LKB1 gene transcription through interaction with their cognate recognition site 5′-GTAAACAA-3′ [22]. FOXO transcription factors become inactivated by certain growth factors [23] through direct phosphorylation by the protein kinase B (PKB) [24], [25]. Since several FOXO target genes are involved in growth control and cell cycle regulation, their inactivation could represent a critical event in malignant transformation [26], [27]. Therefore, our findings that functionally link FOXO proteins to the transcriptional activation of the tumour suppressor gene LKB1, provide an important step towards a detailed understanding of the complex molecular events that promote carcinogenesis.

## Results

### Identification of the LKB1 core promoter

**Figure 1:**
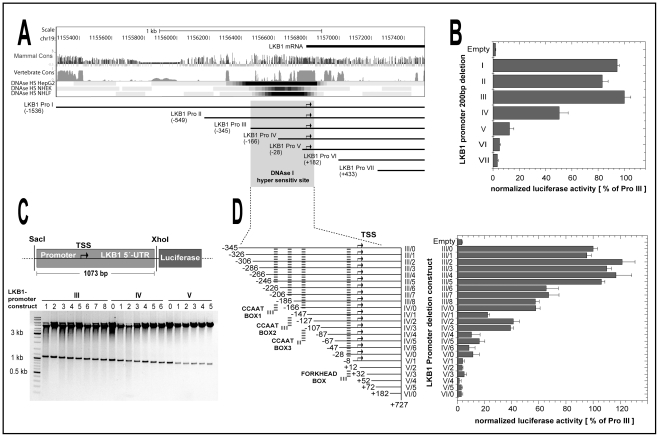
Deletion-mutant analysis of the LKB1 promoter. (**A**) Regions of high mammalian and vertebrate sequence conservation in the 5′-flanking region of the LKB1 coding sequence are aligned to DNase I hypersensitive sites (DNAse HS) in HepG2, normal human epidermal keratinocytes (NHEK) and normal human lung fibroblasts (NHLF) and to LKB1 200 bp deletion mutants (LKB1 Pro I–VII) extending from nucleotide position −1536 to +727 relative to the transcription start site (modified from the UCSC genome browser at http://genome.ucsc.edu/ENCODE/). (**B**) Luciferase activity of transiently transfected “444” cells with 200 bp deletion constructs (I–VII) of the LKB1 promoter. (**C**) 20 bp deletion mutants of the LKB1 promoter Pro III fragment, starting from nucleotide −345 to +727 (III–V) were constructed, digested by the restriction enzymes SacI and XhoI and separated in a 1% agarose gel. The transcriptional start site (TSS) as well as the 5′-untranslated region (5′-UTR) is indicated. (**D**) Comparison of luciferase activity of transiently transfected “444” cells with LKB1 promoter 20 bp deletion constructs (right) and predicted *cis*-regulatory elements (left). The positions of the potential CCAAT boxes I–III as well as the forkhead box are indicated. Luciferase activity of all deletion constructs (relative light units normalized against renilla luciferase activity) is expressed as the percentage of the signal obtained with the plasmid containing the LKB1 promoter region downstream of nucleotide −345 (LKB1 Pro III). All assays were performed three times in quadruplicate. The error bars denote mean ± standard deviation.

As a first step towards localizing important control regions that activate LKB1 gene expression, we cloned the region flanking the 5′-end of the coding sequence in front of a luciferase reporter gene. The reporter plasmid containing the nucleotide sequence from −1536 to +727 relative to the LKB1 transcription start site (referred to as LKB1 Pro I), was active following transient transfection of “444” cells. As a second step, six 200 bp 5′-deletion mutants (referred to as LKB1 Pro II–VII) were constructed (Figure 1A) and luciferase activity was measured (Figure 1B). Consecutive deletion of the sequence from −1536 to −345 resulted in minimal changes of the promoter activity. Stronger decreases were only observed when the LKB1 Pro III construct was further deleted, indicating that important *cis*-regulatory elements are located in the area downstream of nucleotide −345. The deletion from −345 to −186 (LKB1 Pro IV) reduced luciferase activity by 50%. The next shorter deletion construct (LKB1 Pro V) showed only 12.5% of the LKB1 Pro III activity. Removing the transcriptional start site [3] by further truncation of LKB1 Pro V, decreased luciferase activity to a level similar to that obtained by transfection with the empty vector. The fact that the residual 550 bp of the 5′-untranslated region (5′-UTR) within LKB1 Pro VI could not activate reporter gene expression alone, indicates that important regulatory elements are located upstream of nucleotide +182.

Interestingly, data-base analysis using the human genome browser of the Encyclopedia of DNA Elements (ENCODE) [28] revealed that the area between nucleotide −345 and the transcriptional start site coincides with a previously described DNaseI hypersensitive region (Figure 1A). This has been identified by a genome-wide chromatin analysis in different cell types including HepG2 cells, normal human epidermal keratinocytes and normal human lung fibroblasts [29]. Since the sequence within the LKB1 Pro III construct is crucial for reporter gene transcription and co-localize with a nucleosomal structure that is highly accessible to exogenously added DNaseI, favours the notion that the LKB1 core promoter is located downstream of nucleotide −345.

### Deletion mutant analysis of the LKB1 promoter identifies regulatory regions

In order to localize critical *cis*-regulatory elements within the LKB1 core promoter more precisely, we constructed a series of 20 bp deletion mutants of LKB1 Pro III (Figure 1C). These constructs (III/0–8; IV/0–6 and V/0–5) are schematically outlined in Figure 1D (left). After transient transfection of “444” cells with these 24 different deletion constructs, including those used in the previous assay (LKB1 Pro III/0, IV/0, V/0 and VI/0, see Figure 1A), luciferase activity was measured (Figure 1D, right). While sequence deletions from −345 to −246 did not affect promoter activity, further truncation of III/5 to III/6 reduced luciferase activity to 65% of the wild-type promoter. The next drop in luciferase activity was observed between III/7 and III/8 when nucleotides −206 to −187 were deleted. III/8 and IV/0 reached only 57% of the LKB1 Pro III/0 reporter activity and it even dropped to 22% after removing nucleotides −166 to −147. Interestingly, all three sequence deletions between nucleotides −345 and −147 that decreased LKB1 promoter activity exhibit homology to consensus sequences for CCAAT boxes [30] (Figure 1D, left). Another critical deletion removes a sequence homologous to a transcription factor consensus site was located between nucleotides −28 and −8 (Figure 1D, left). It contains a potential binding site for FOXO transcription factors [22] and reduced luciferase activity of the V/0 construct by 65%. The residual activity was similar to that of the promoterless vector (Figure 1D, right).

Likewise, deletions of each CCAAT box and the predicted FOXO binding site also decreased luciferase activity when HPV-negative C33a cells were transfected, although the extent of reduction was slightly different (Figure S1). Two deletions were found to have effects on promoter activity, which were apparently cell type specific. While “444” cells showed an additional drop in activity between IV/3 and IV/4, activity in C33a cells decreased between IV/4 and IV/5.

### Substitution mutant analyses identify LKB1 promoter elements critical for activated transcription

**Figure 2:**
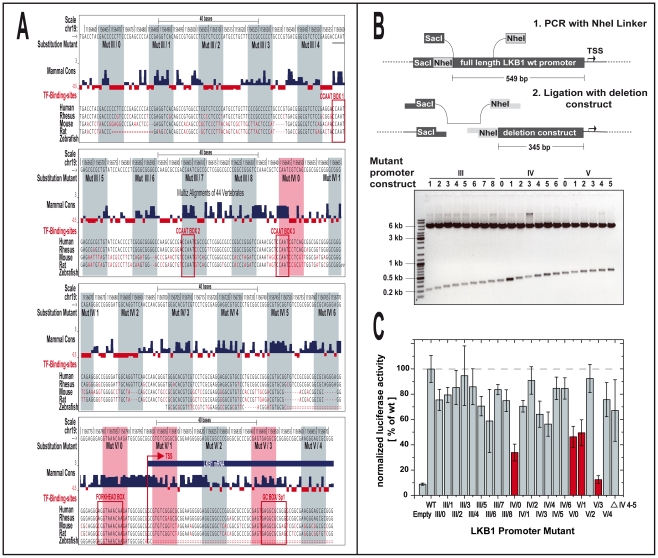
Substitution mutant analysis of the LKB1 promoter. Match of phylogenetic footprint with substitution mutant analysis of the LKB1 promoter region ranging from nucleotide position −345 to +75. (**A**) Mammalian base-wise conservation (blue and red bars) is displayed together with sequence alignments of different species, predicted transcription factor (TF)-binding sites (red boxes) and positions of mutations within the corresponding substitution mutants of the LKB1 promoter reporter constructs. Conserved sequences (“Mammal Cons”) are indicated in blue, non-conserved sequences in red. The height of the corresponding bars represents the degree of conservation. Mutations which reduce reporter activity in the substitution mutant analysis by more than 50% are highlighted in a light red background, while mutations without influence on reporter activity are coloured in a light blue background. Display of alignments and mammalian conservation were modified from the UCSC genome browser (see Fig. 1). The positions of the potential CCAAT/Forkhead/Sp1 boxes are indicated as red rectangles. (**B**) Substitution mutants of the LKB1 promoter Pro II fragment, encompassing nucleotides −549 to +727, were constructed by introducing a 10 bp mutation, comprising a NheI restriction site, within the area of −345 to +75 by PCR and religation with the respective deletion mutant. The presence of the mutation within the LKB1 promoter was monitored by agarose gel electrophoresis following restriction enzyme digestion with SacI and NheI. (**C**) Luciferase activity of transiently transfected “444” cells with LKB1 promoter 10 bp substitution mutants reveals a critical role of four *cis*-acting elements regulating LKB1 transcription (red bars). Activity of substitution mutants (relative light units normalized against renilla luciferase activity) is expressed as the percentage of the signal obtained with the plasmid containing the LKB1 wild-type promoter (LKB1 Pro II, −549 to +727). Each bar represents the means ± standard deviation of three independent experiments made in quadruplicate.

In the course of our 20 bp deletion mutation analysis, we found strong promoter activity within the area downstream of nucleotide −345 of the LKB1 promoter which is apparently mediated by CCAAT boxes and a FOXO binding site (Figure 1D, left). Interestingly, phylogenetic footprint analysis revealed high homology of these elements between human, rhesus, mouse, rat and zebrafish, indicating that the sequences required for the binding of activators to the promoter have been conserved, despite having evolved under heterogeneous constraints (Figure 2A). In order to confirm the relevance of these regions in a context in which the promoter length remains unchanged, we generated 21 substitution mutants where 10 bp sequences between positions −345 to +72 were systematically altered by inserting a NheI restriction site (Figure 2B, referred to as III/1–8, IV/0–6 and V/0–5). After transient transfection of “444” and C33a cells with the mutant promoter constructs, luciferase activity was measured (Figure 2C, “444”; Figure S1, C33a) and the result was compared to the phylogenetic footprint analysis of the −345 to +72 sequence (Figure 2A). The first potential CCAAT box, whose deletion had reduced promoter activity by 35%, was not disrupted by the corresponding substitution mutation III/5. Mutation of the second CCAAT box (III/7), substituting nucleotides −196 to −187, caused only a minimal decrease of 16% in luciferase activity in “444” cells. Consistent with the previous analysis (Figure 1D), in which deletion of the site caused only 12% reduction in activity, this suggests a less important function of the second CCAAT box.

The first clear reduction that reduced luciferase activity in both cell lines investigated was generated by mutation of the sequence from −157 to −148, containing the third potential CCAAT box. The corresponding construct (IV/0) showed only 34% of the wild-type activity in “444” cells. Another critical element was disrupted by the V/0 mutation, which reduced wild type activity to 46% in “444” cells. This element contains the potential FOXO binding site and did also affect promoter activity in the deletion analysis (Figure 1D, right). In addition, we found two other mutations downstream of the FOXO site that reduced promoter activity by greater than 50% in both cell lines. One was disrupting the area around the transcription start site from +3 to +12 where the corresponding construct (V/1) showed 49% of the wild-type activity in “444” cells. The other mutation was disrupting an element that was most critical for promoter activity. Notably, this element was located within the 5′-UTR between nucleotides +43 to +52. The corresponding mutant (V/3) showed only 10% of the wild-type activity (Figure 2C, marked in red) and lacks apparent similarities to consensus sites for known transcription factors.

### The transcription factor Sp1 binds to a regulatory element downstream of the transcriptional start site

**Figure 3:**
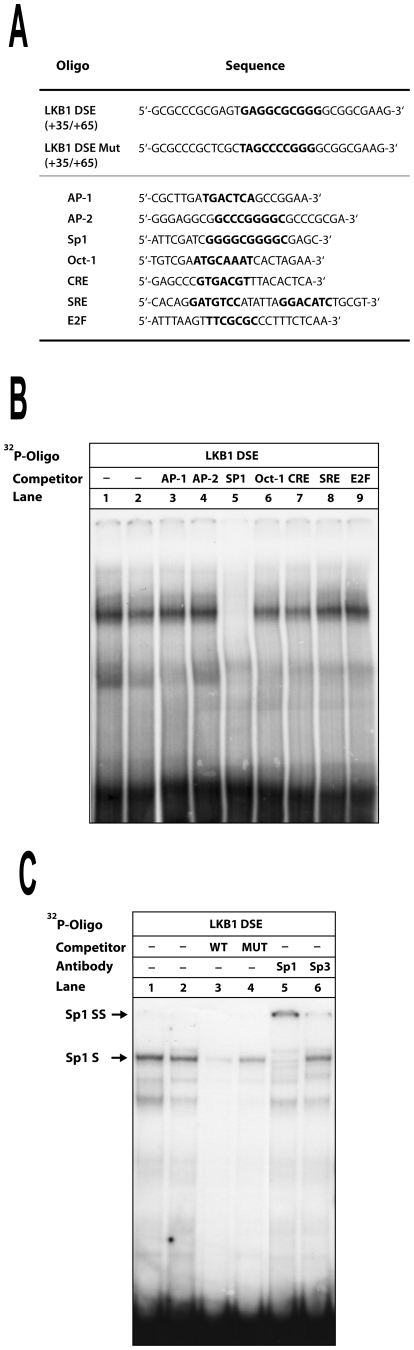
Binding of Sp1 to a non-canonical GC box downstream of the transcription start site. (**A**) Overview of oligonucleotides used for EMSA. LKB1 DSE: downstream element, position +35 to +65 within the LKB1 5′-UTR and the corresponding mutant (LKB1 DSE Mut). Sequences of oligonuceotides containing consensus sites of different transcription factors used for competition are given below. (**B**) The radioactively labeled LKB1 DSE was incubated with 4 µg of nuclear extracts obtained from “444” cells (lane 1) or HepG2 cells (lane 2–9). A 500-fold molar excess of indicated unlabeled competitor oligos was added (lanes 3–9) and separated in a 7% polyacrylamide gel. (**C**) Complex formation (Sp1 S) of the same probe with 4 µg of nuclear extracts from “444” cells (lane 1) or from HepG2 cells (lane 2–6) was self-competed by a 500-fold molar excess of wild-type oligo (WT; lanes 3) but not by a mutant oligo (LKB1 DSE MUT; lane 4). Addition of an antibody against Sp1 (sc-59 X) (lane 5) retarded the specific complex (Sp1 SS) while addition of an antibody against Sp 3 (lane 6) did not.

To get insight which transcription factor can bind to this element within the 5′-UTR, electrophoretic mobility shift assays (EMSA) were performed. When nuclear extracts of “444” and HepG2 cells were incubated with a DNA probe comprising this LKB1 downstream element (LKB1 DSE), a single protein-DNA complex was observed (Figure 3B, lanes 1–2). To characterize the nature of the corresponding binding factor, we used an excess of different unlabeled competitor oligonucleotides containing consensus sequences for known transcription factors (Figure 3A). While the intensity of the band was not affected by the addition of competitor containing AP-1, AP-2, Oct-1, CRE, SRE and E2F sites (Figure 3B, lanes 3–4 and 6–9, respectively), binding was completely inhibited by an excess of a Sp1 consensus site containing oligonucleotide (lane 5). Furthermore, complex formation was competed by a molar excess of the wild-type oligo, but not by the corresponding mutant oligo harbouring the same mutations as the substitution mutant V/3 (Figure 3C, lanes 3 and 4, respectively). Finally, addition of an antibody against the transcription factor Sp1 specifically retarded the complex, while the addition of an antibody against the closely related Sp3 did not (Figure 3C, lanes 5 and 6). Although the LKB1 DSE within the 5′-UTR differs from the classical GC box at two nucleotide positions, a similar binding site for Sp1 has been identified previously [31]. These results suggest that the ubiquitously expressed transcription factor Sp1 is involved in LKB1 regulation.

### Binding of NF-Y to three CCAAT boxes within the LKB1 promoter

**Figure 4:**
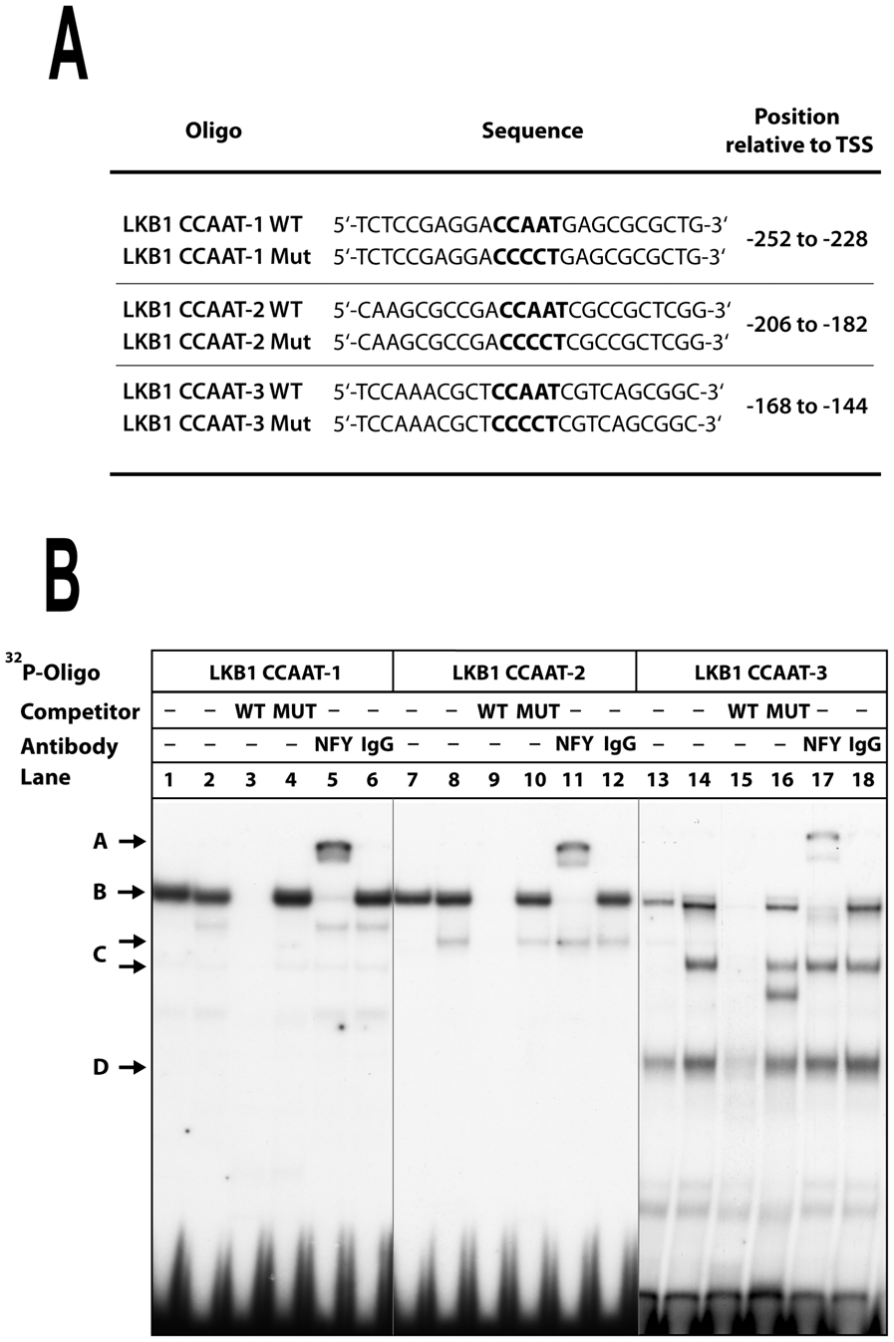
Binding of NF-Y transcription factor to CCAAT boxes within the LKB1 promoter. ^32^P-labeled double-stranded oligonucleotides harbouring the CCAAT boxes (their positions relative to the transcriptional start site, TSS, are indicated) (**A**) were incubated with 2 µg of nuclear extracts from “444” cells (lanes 1, 7 and 13) or from HepG2 cells (lanes 2–6, 8–12, and 14–18) and separated in a 7% polyacrylamide gel (**B**). Formation of sequence specific protein complexes was confirmed by competition with unlabeled oligos. Specific bands (indicated by arrows B, C and D) were competed by a 500-fold molar excess of wild-type oligo (WT; lanes 3, 9 and 15) but not by the same excess of mutant oligo (MUT; lanes 4, 10 and 16, respectively). Protein complexes containing the transcription factor NF-Y (arrow A) were further retarded by addition of an antibody against NF-Yα (NFY; lanes 5, 11 and 17) but not by the addition of the same amount of normal goat IgG (IgG; lanes 6, 12 and 18).

Based on the results of the LKB1 promoter mutant analysis, oligonucleotides containing the potential CCAAT boxes were also examined by EMSA (Figure 4A). Nuclear extracts from “444” and HepG2 cells were incubated with three different radiolabeled double-stranded oligonucleotides (LKB1 CCAAT-1, 2 and 3, Figure 4B). Both extracts generated one predominant protein-DNA complex with all three oligonucleotides (“complex B”). In addition, both cell lines formed a second complex (complex D) only with the LKB1 CCAAT-3 oligo. Another complex (“complex C”) was only formed in HepG2 cells when oligos CCAAT-2 and 3 were examined. Specificity was confirmed by the absence of complex formation when a 500-fold molar excess of the unlabeled wild-type oligo was included (lanes 3, 9 and 15, respectively). In contrast, competition with a mutant oligo had no effect on complex formation (lanes 4, 10 and 16).

The heterotrimeric transcription factor NF-Y has been described to bind CCAAT boxes [30]. To test whether these protein-DNA complexes contain NF-Y proteins, antibody incubation experiments were performed. Indeed, a polyclonal antibody directed against the alpha subunit of NF-Y altered the mobility of the uppermost complex (“complex A”; lanes 5, 11 and 17, respectively) in comparison to the addition of a non-specific antibody (lanes 6, 12 and 18). Notably, the HepG2 specific “complex C” was not shifted by the NF-YA antibody, indicating that an additional protein, which is not present in “444” cells can bind to this element. Due to a different metabolic state, it is possible that the HepG2 specific complex may be necessary for the high transcriptional steady-state level of the LKB1 gene in liver cells [3].

### Interaction between FOXO proteins and the LKB1 promoter

**Figure 5:**
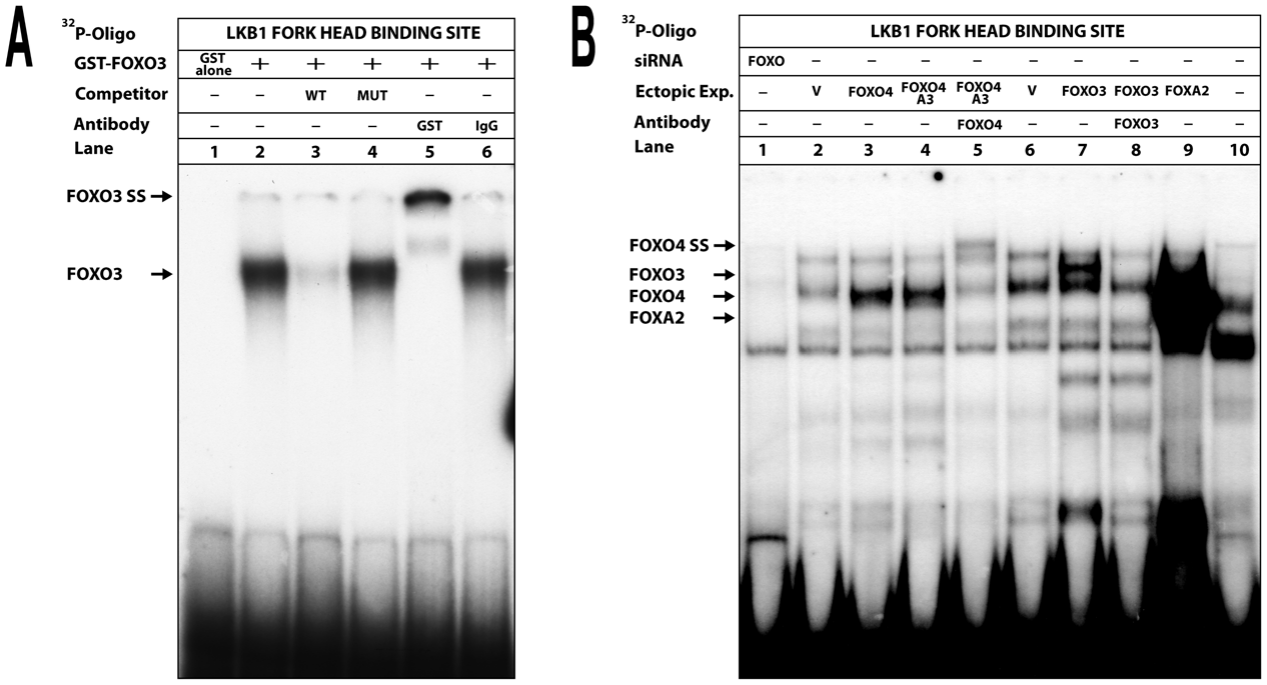
Binding of forkhead box transcription factors FOXO3 and FOXO4 to the LKB1 promoter. (**A**) The ^32^P-labeled double-stranded oligonucleotide 5′-GGGGAGGGAGGTAAACAAGATGGCGGC-3′ containing the −28 to −2 region of the LKB1 core promoter was incubated with either 25 ng of recombinant GST (lane1) or GST-tagged FOXO3 protein (lane 2–6) and separated in a 4% polyacrylamide gel. (**B**) The same oligonucleotide as described in (A), but incubated with 4 µg of nuclear extracts from “444” cells (lanes 1–9) or from HepG2 cells (lane 10) in the presence of a 500-fold molar excess of the mutant unlabeled oligo 5′-GGGGAGGGAGGTAGCCAAGATGGCGGC-3′. Protein complexes containing the transcription factors FOXO3, FOXO4 and FOXA2 are indicated by arrows. Cells were transfected with either siRNA against the FOXO family members (lane1) or with expression plasmids encoding FOXO4 (lane 3), mutant FOXO4 A3 (lane 4 and 5), FOXO3 (lane 7 and 8), FOXA2 (lane 9) or with the corresponding empty vectors (V) (lane 2 and 6). Addition of an antibody against FOXO4 (lane 5) resulted in further retardation of the FOXO4 containing complex (FOXO4 SS), while addition of the FOXO3 antibody (lane 8) inhibited formation of the FOXO3 complex.

Since mutation of a potential forkhead box transcription factor binding site within the LKB1 promoter significantly reduced promoter activity (Figure 1D), we assumed that FOXO proteins can bind to this element. To test this assumption, EMSA using a radiolabeled double-stranded oligonucleotide, containing the potential FOXO binding site of the LKB1 promoter was carried out (Figure 5). Purified GST-tagged FOXO3 protein (lanes 2–6), but not GST interacted specifically with the oligo (lane 1) resulting in the formation of a single DNA-protein complex (Figure 5A). Sequence specificity of the complex was further confirmed by oligonucleotide competition and antibody incubation experiments. Here, complex formation was both effectively competed by an excess of unlabeled wild-type but not with mutant oligo and supershifted by the addition of a GST specific antibody.

In further experiments, nuclear extracts from “444” cells after transfection with either expression plasmids encoding different FOXO proteins or siRNA directed against all FOXO factors in non-transfected cells were incubated with the probe in the presence of an excess of mutated competitor (Figure 5B). Complex formation was inhibited in cells treated with siRNA against FOXO proteins (lane 1). In contrast, ectopic expression of FOXO4 (lane 3) or mutant FOXO4 A3 (lane 4) that is constantly localized within the nucleus [32], as well as FOXO3 (lane 7) increased formation of the FOXO containing complexes when compared to transfections with the corresponding empty vectors (indicated a “V”, see lanes 2 and 6). Moreover, an antibody against FOXO4 altered the mobility of the FOXO4 containing complex (FOXO4 supershift, SS; lane 5) and an antibody against FOXO3 disrupted the FOXO3 containing complex (lane 8). In addition, incubation of the oligonucleotide with extracts from HepG2 cells resulted in the formation of a different complex (lane 10). It is unlikely that this complex is formed by another FOXO member, since none of the used cell lines expressed FOXO1 or FOXO6 (data not shown). Since HepG2 cells express the liver specific transcription factor FOXA2, also known as HNF-3β [33], it can be assumed that the observed protein-DNA complex may contain this factor. After ectopic expression of FOXA2 in “444” cells, known to lack this factor endogenously, complex formation with similar mobility could be discerned (lane 9), indicating that also other forkhead box transcription factors can bind to this element in a tissue specific manner. However, as deduced from EMSA analysis, FOXO3 and FOXO4 seem to be the key player in LKB1 gene regulation (see below).

### Chromatin immunoprecipitation demonstrates binding of NFY, Sp1 and FOXO proteins to the endogenous LKB1 promoter

**Figure 6:**
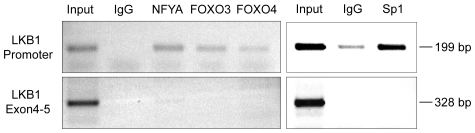
Chromatin immunoprecipitation (ChIP) analysis of NF-Y, FOXO3 and FOXO4 binding at the LKB1 promoter. Enrichment of PCR products specific for the LKB1 promoter region (upper panel) in comparison to PCR products specific for the LKB1 coding region encompassing exon 4–5 (lower panel). PCR products were amplified from sonified DNA after ChIP, subsequently run on a 1% agarose gel and stained with ethidium bromide. As a positive control (Input), 1/10 of the starting material was used for PCR. Protein-DNA complexes were either incubated with non-specific goat IgG (negative control) or with antibodies against the transcription factors NFY-α, Sp1 (sc-14027 X), FOXO3 or FOXO4. The figure shows a representative of three independent experiments.

In order to examine whether NF-Y, Sp1 and the FOXO proteins also bind to the LKB1 promoter *in vivo*, chromatin immunoprecipitation (ChIP) assays were performed (Figure 6). All antibodies against NF-YA, Sp1, FOXO3 and FOXO4 specifically enriched the region containing the LKB1 core promoter in comparison to a non-specific antibody. In contrast, no enrichment was observed for the LKB1 coding sequence. Taken together, the ChIP analysis confirms the interaction between NF-Y, FOXO3 and FOXO4 with the endogenous LKB1 promoter and further supports a critical role of these factors in LKB1 gene expression.

### Ectopic expression of Sp1, NF-Y and the FOXO transcription factors FOXO3 and FOXO4 activates the LKB1 promoter

**Figure 7:**
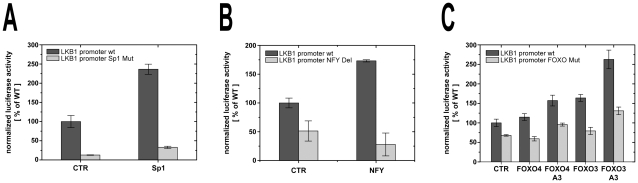
NF-Y, Sp1 and FOXO transcription factors activate transcription from the LKB1 promoter. Luciferase reporter assays in C33a cells after over-expression of Sp1 (**A**), NF-Y (**B**) and FOXO transcription factors (**C**). Reporter activity of LKB1 wild-type promoter is indicated in dark grey, while reporter activity of constructs lacking the corresponding transcription factor binding site is indicated in light grey. Luciferase activity (relative light units normalized to renilla luciferase activity) is expressed as the percentage of the signal obtained from co-transfection of 100 ng of the plasmid containing the LKB1 wild-type promoter (LKB1 Pro II, −549 to +727) together with 150 ng of the empty expression vector (CTR). Instead of the empty vector 150 ng of the corresponding transcription factor expression plasmid have been co-transfected. In the case of NF-Y 50 ng of plasmids encoding each subunit NF-Yα, NFY-β and NF-Yγ were transfected together. Each bar represents the means ± standard deviation of three independent experiments.

In order to show a function of these factors in LKB1 regulation, co-transfections with LKB1 luciferase reporter constructs and expression plasmids encoding the different transcription factors were performed (Figure 7). Transient transfection of Sp1 significantly increased luciferase activity of the wild-type promoter when compared with the empty vector. In contrast, no induction could be discerned when Sp1 was co-transfected with the substitution construct (LKB1 Pro V/3) where the Sp1 binding site was mutated (Figure 7A). Ectopic expression of all three NF-Y subunits also activated the LKB1 wild-type promoter, while the deletion construct that lacks all three CCAAT boxes (LKB1 Pro IV/1) could not be induced (Figure 7B). Finally, FOXO proteins also induced reporter gene expression in a sequence specific manner, since the respective mutant LKB1 Pro Mut V/0 was not activated to the same extent after co-transfection with different FOXO expression plasmids (Figure 7C). Although FOXO3 expression significantly increased luciferase activity of the wild-type promoter, induction after FOXO4 transfection was only marginal. One reason for this could be a post-translational modification of the FOXO4 protein. As described above, transcriptional activity of all FOXO proteins is negatively regulated by the protein kinase B (PKB) through direct phosphorylation of three amino acid side chains [24], [25]. When mutants of the two FOXO proteins, which lack these PKB phosphorylation sites (FOXO3 A3 and FOXO4 A3) were transfected, LKB1 reporter gene expression was activated even more efficiently (Figure 7C). These results demonstrate that Sp1, NF-Y as well as FOXO3 and FOXO4 have the ability to activate the LKB1 promoter through interaction with their corresponding binding sites.

### Knockdown of NF-Y and FOXO transcription factors inhibits LKB1 gene expression

**Figure 8:**
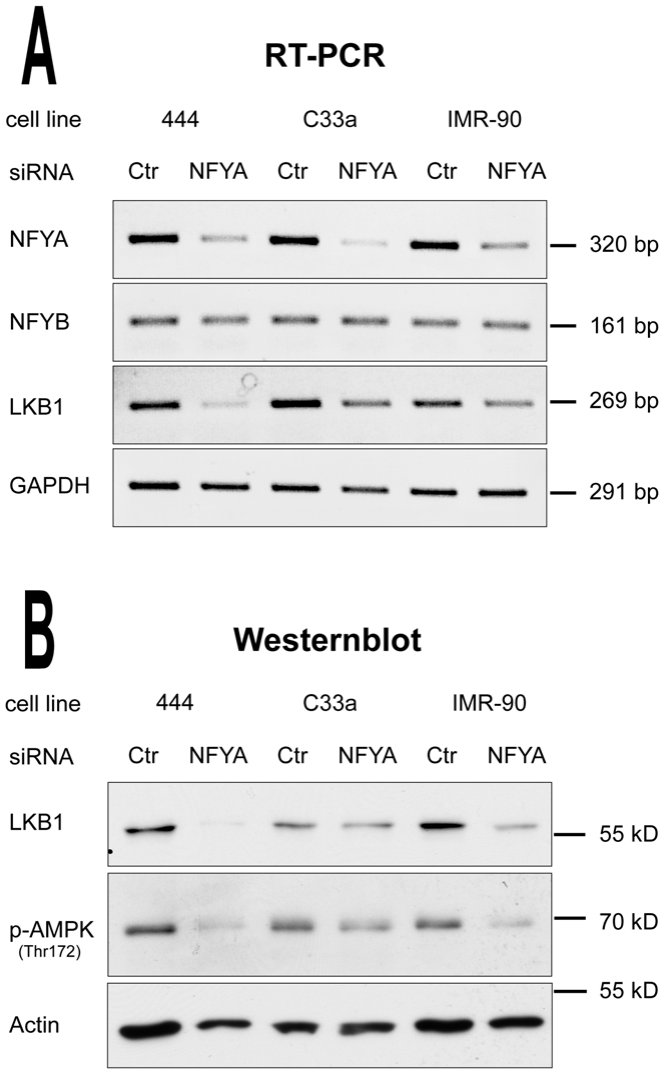
NF-Yα transcription factor is required for LKB1 gene expression. Knockdown of endogenous NF-Yα by siRNA inhibits LKB1 expression in “444”, C33a and IMR-90 cells. Cells were transfected either with scrambled siRNA (Ctr) or with a siRNA targeting NF-Yα (NF-YA). Total RNA was purified and mRNA levels of NF-YA, NF-YB, LKB1 and GAPDH were analysed by RT-PCR using ethidium bromide staining (**A**). Equal amounts of total protein (5 µg) were separated by SDS-PAGE and analysed by western blotting using antibodies against LKB1, phospho-AMPK (Thr172) and actin (**B**). The data shown is representative of three independent experiments.

**Figure 9:**
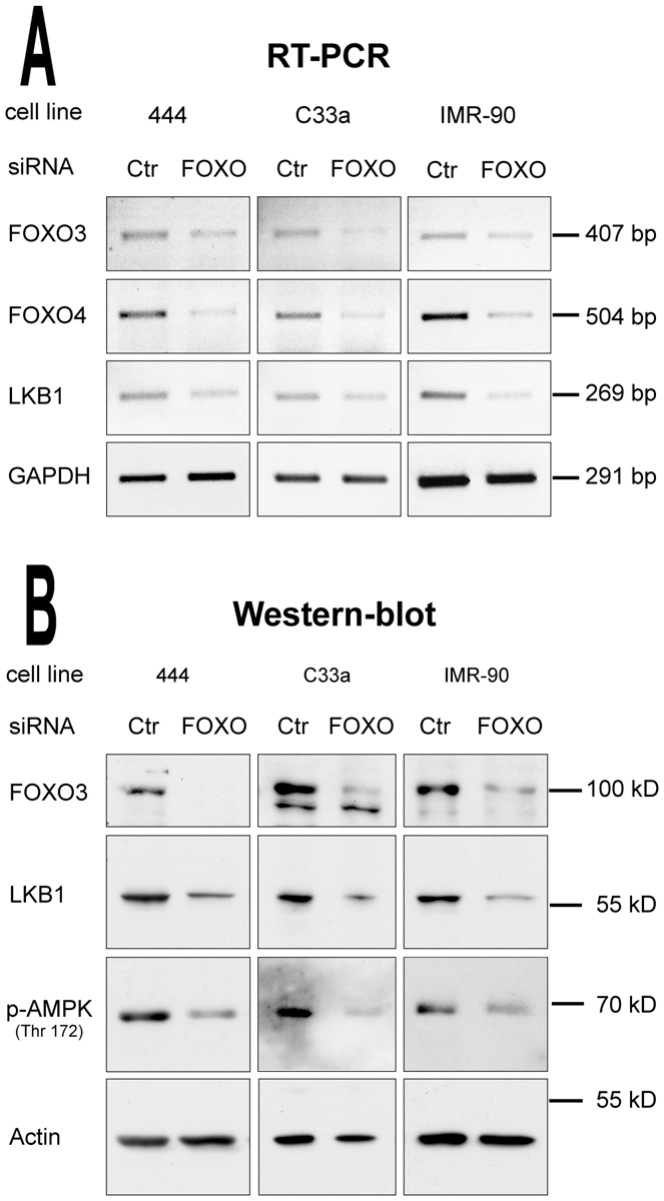
FOXO transcription factors are required for LKB1 gene expression. Knockdown of endogenous FOXO3 and FOXO4 in “444”, C33a and IMR-90 cells. Cells were transfected either with scrambled siRNA (Ctr) or with a siRNA targeting all FOXO family members (FOXO). Total RNA was purified and relative mRNA levels of FOXO3, FOXO4, LKB1 and GAPDH were analysed by RT-PCR using ethidium bromide staining (**A**). Equal amounts of total protein (5 µg) were separated by SDS-PAGE and analysed by western blotting using antibodies against FOXO3, LKB1 phospho-AMPK (Thr172) and actin (**B**). The data shown is representative of three independent experiments.

To further investigate the role of NF-Y and the FOXO proteins in activating endogenous LKB1 gene transcription, siRNA knockdown experiments were performed. While delivery of siRNA against Sp1 resulted in massive cell death, treatment of “444”, C33a and IMR-90 cells with NF-YA siRNA significantly diminished endogenous mRNA levels of the alpha subunit of the NF-Y complex (Figure 8A). In contrast, mRNA levels of NF-YB and GAPDH, which were used as internal controls, were not affected. Consistent with the previous experiments, LKB1 mRNA and protein levels were strongly reduced after the NF-YA knockdown, indicating that NF-Y is essential for LKB1 gene expression (Figure 8A and B). Moreover, siRNA knockdown of FOXO3 and FOXO4 also decreased LKB1 expression, both on mRNA and protein level (Figure 9). Furthermore, phosphorylation of AMPKα, the main substrate of LKB1 was also significantly reduced under these conditions (Figure 8 and 9), indicating that individual knockdown of both NF-YA or the two FOXO proteins FOXO3 and FOXO4 not only diminished LKB1 gene expression, but also interfered with the LKB1-AMPK signalling pathway by reducing AMPK activation.

## Discussion

In the present study we performed a systematic analysis of the LKB1 promoter. Using extensive deletion and mutant analyses, we identified multiple control elements important for LKB1 gene transcription. Three of these elements contained CCAAT boxes and were recognized by the heterotrimeric transcription factor NF-Y [30]. Another evolutionary highly conserved element contained a binding site for forkhead box transcription factors and interacted specifically with the transcription factors FOXO3 and FOXO4 [22].

Besides these factors, whose binding sites were all located upstream of the transcription start site, we found another critical element within the 5′-untranslated region (5′-UTR). It has been identified as a Sp1 site that was apparently essential for promoter activity in the transient transfection assays (Figure 2). Furthermore, ectopic expression of Sp1 significantly increased LKB1 promoter activity (Figure 7A).

Although Sp1 was longtime considered to be a house-keeping transcription factor, the protein is involved in glucose metabolism where Sp1 binding to the acetyl-CoA carboxylase promoter increased transcription in adipocytes [34]. Moreover, Sp1 is also regulated by energy deprivation [35] and plays a role in insulin signaling. However, to which extent Sp1 contributes to endogenous LKB1 expression still remains to be elucidated, since siRNA knockdown of Sp1 caused massive cell death in all cell lines investigated. Remarkably, the Sp1 element was not sufficient to activate reporter gene expression alone (Figure 1). It is therefore tempting to speculate that the Sp1 site located within the 5′-UTR apparently may cooperate with control elements located upstream of the transcription start site. Indeed, a precedential case has been described for the major histocompatibility complex class II-associated invariant chain, where Sp1 and NF-Y cooperatively activate the gene in cancer cell lines [36]. Consistent with this notion is the fact that siRNA knockdown of the alpha subunit of NF-Y but also of FOXO transcription factors greatly reduced endogenous LKB1 expression (Figure 8 and 9). Moreover, phosphorylation of the alpha subunit of AMPK, the main downstream effector of LKB1, was also diminished under these conditions. This indicates that these proteins, in conjunction with Sp1, are not only indispensible for efficient transcription of the gene, but also affect the activation of downstream targets in the LKB1 signaling pathway.

Although we have shown that Sp1, NF-Y and the FOXO transcription factors are necessary for effective LKB1 gene transcription in our cell systems, there also exists the possibility that other functionally important *cis*-regulatory elements and *trans*-acting factors exist that influence LKB1 promoter activity in a tissue-specific manner. First, deletion and substitution mutations at other locations had cell line specific but significant effects on promoter activity (Comparison Figure 1 and Figure S1). Second, other cell line specific DNA-binding activities were detected by EMSA. In detail, the liver specific transcription factor FOXA2 (also named HNF-3β) was also able to bind to the FOXO recognition site in the LKB1 promoter (Figure 5). Furthermore, additional studies have shown that different members of this huge family of transcription factors exhibit nearly identical binding motifs [37], [38]. It therefore remains possible that beside FOXO3 and FOXO4, also other members of this transcription factors family may regulate LKB1 gene expression in a cell type specific manner. Since the expression pattern of each family member ranges from restricted to a single tissue to nearly ubiquitous, it also seems possible that these factors are either redundant or indispensable in certain tissues. Further experiments will be needed to dissect the role of other forkhead box transcription factors in the regulation of the LKB1 promoter in various cell types and in correlation with their metabolic state.

Notably, the activity of FOXO transcription factors is not only controlled by tissue specific expression, but also by a variety of posttranslational modification, including phosphorylation, acetylation and ubiquitination [23]. For instance, phosphorylation of FOXO3 by AMPK leads to the activation of its transcriptional activity [39]. Having shown that LKB1 transcription is induced by FOXO3 and since AMPK is a direct target of LKB1 [12], [13], our analysis suggests that the expression of LKB1 might be stimulated by AMPK via a positive regulatory feedback loop, allowing a cross-talk between these enzymes to counterbalance each other in the coordination of anabolic and catabolic activities.

How could LKB1 react to changes of the external milieu? Here, phosphorylation of three conserved serine/threonine residues within FOXO proteins by the proto-oncogene PKB may play an important role [24], [25]. This specific phosphorylation is mainly triggered by growth factors like insulin or the insulin-like growth factors and results in the inhibition of FOXO factors due to their export from the nucleus [32]. Notably, in our experiments we could show that PKB phosphorylation site deficient mutants of FOXO3 and FOXO4 were even more potent in inducing LKB1 promoter activity than the wild type forms (Figure 7C). It therefore seems possible that the insulin-phosphatidylinositol 3-kinase – PKB signalling pathway negatively regulates LKB1 expression through inactivation of FOXO transcription factors. Although further experiments will be needed to prove this assumption, there are other reports supporting the existence of this functional link. Recently it was shown that LKB1 transcription was down-regulated upon induction of the serum and glucocorticoid-inducible kinase 1 (SGK-1) [40]. SGK-1, like PKB, belongs to the same family of protein kinases [41], is a downstream effector of the phosphatidylinositol 3-kinase pathway and is also able to phosphorylate FOXO transcription factors at the same residues like PKB [42]. Since there is now considerable evidence that insulin and the insulin-like growth factors play important roles in neoplasia [43], it will be one of the future goals to elucidate whether these hormones are involved in the down regulation of the LKB1 tumour suppressor in certain tumour entities.

## Materials and Methods

### Antibodies

All antibodies were obtained from commercial suppliers and used without further purification. Mouse monoclonal anti-LKB1 (ab15095) was purchased from Abcam, rabbit monoclonal anti-phospho-AMPKα Thr172 (2535/40H9) was purchased from Cell Signaling Technology, goat polyclonal anti-NF-YA (sc-7712 X), rabbit polyclonal anti-FOXO3 (sc-11351 X), goat polyclonal anti-FOXO4 (sc-5221 X), rabbit polyclonal anti-Sp1 (sc-59 X), rabbit polyclonal anti-Sp1 (sc-14027 X), rabbit polyclonal anti-Sp3 (sc-644 X), rabbit polyclonal anti glutathione-S-transferase (GST) (sc-459) and normal goat IgG (sc-2028) were from Santa Cruz Biotechnology. Horseradish peroxidase-conjugated secondary antibodies, polyclonal goat anti-rabbit IgG (W4011) and anti-mouse IgG (W4012) were from Promega.

### Cell Culture

The HPV-negative cervical carcinoma cell line C33a and hepatocellular carcinoma cells HepG2 were obtained from American Type Culture Collection. The normal fibroblast line IMR-90 and the non-tumourigenic somatic cell hybrids made between HeLa cells and IMR-90 (referred to as “444”) were kindly provided by E. Stanbridge [44], [45]. All cells were maintained in Dulbecco modified Eagle medium (DMEM) (Sigma) supplemented with 10% (v/v) of foetal bovine serum (Gibco Life Technologies, Paisley, United Kingdom), penicillin (final concentration: 100 U/ml, Gibco) and streptomycin (final concentration: 0.1 mg/ml, Gibco).

### Plasmids

The plasmids pJET1.2 (Fermentas), pGL3-Basic (Promega), pRL-null (Promega), pRL-TK (Promega) and pcDNA3 (Invitrogen) have been purchased from commercial suppliers.

The 5′-flanking region of the LKB1 coding sequence encompassing nucleotides −1536 to +1321 relative to the transcription start site was amplified by PCR using 200 nM of forward (5′-CACCCTGCCTAATGTCCCTA-3′) and reverse (5′-GAGTCCAGCACCTCCTTCAC-3′) primers, 2 ng/µl of genomic DNA from “444” cells as a template and 0.05 U/µl of PfuUltra HF DNA polymerase (Stratagene) in a final volume of 25 µl PfuUltra reaction buffer containing 0.1 mM dNTP's, 4% of dimethylsulfoxide and 2% of formamide. The PCR-product was purified, cloned into the pJET1.2 plasmid using the CloneJET PCR Cloning Kit (Fermentas) according to the manufacturer's instructions and verified by DNA sequencing. The LKB1 promoter (position −1536 to +727) was then subcloned into the SmaI site of the pGL3-Basic luciferase reporter vector by PCR amplification using the same forward primer and the reverse primer (5′-GCCCACGGACAAGTATGAAC-3′) with Phusion High-Fidelity DNA-polymerase (Finnzymes) according to the manufacturer's instructions. Deletion mutants were generated by PCR using primers listed in Table S1 and inserted into the SmaI site of the pGL3-Basic plasmid. Substitution mutants of the LKB1 promoter were derived from the deletion mutants by inserting a second PCR product expanding from −549 to the deletion end point, using an upstream primer containing a SacI site and the −549 sequence and downstream primers containing a NheI site and the sequence adjacent to the respective deletion end point (Table S1).

The renilla luciferase reporter construct pRL-TATA was constructed by inserting a minimal TATA box of the adenovirus type 2 major late promoter [46] between the BglII and SalI sites of the pRL-null plasmid.

Eukaryotic expression plasmids were generated by inserting PCR amplified full length cDNA of NF-YA (NM_002505.4), NF-YB (NM_006166), NF-YC (NM_014223.4), FOXO3 (BC058662) and FOXO4 (BC106761) into the EcoRV site of the pcDNA3 vector. Primers used for PCR amplification are listed in Table S1. FOXO3 A3 was created by mutating the three Akt/PKB phosphorylation sites T32, S253 and S315 to A and FOXO4 A3 has been generated by site directed mutagenesis of amino acid residues T32, S197 and S262 to A using primers listed in Table S1.

All plasmids used in transient transfections were purified with the QIAGEN Plasmid Maxi Kit (Qiagen) and verified by sequencing prior to transfections.

### Luciferase Assays

One day before transfection, C33a and “444” cells were plated at a density of 2.5×10^4^/well on a white Nunclon F-96-well plate (Nunc, Roskilde, Danmark). For the deletion- and substitution mutant analysis, cells were transfected with 250 ng/well of LKB1-promoter pGL3 firefly luciferase reporter and 25 ng/well of pRL-TK renilla luciferase plasmid for normalization using 0.9 µl/well of Lipofectamine 2000 (Invitrogen) for C33a cells and 1.2 µl/well for “444” cells. 48 h after transfection, cells were lysed, firefly luciferase activity was analysed and normalized to renilla luciferase activity using the dual-luciferase reporter assay system (Promega) according to the manufacturer's instructions. For co-expression of transcription factors and LKB1-luciferase reporters, cells were transfected with 100 ng/well of LKB1-promoter driven pGL3 firefly luciferase reporters, 0.5 ng/well of pRL-TATA normalisation plasmid and 150 ng/well of the corresponding expression plasmid. All experiments were performed at least 3 times in quadruplicates. A one-way analysis of variance (ANOVA) using the GraphPad PRISM® program Version 5.0 followed by a Newman-Keuls post-hoc test was performed for statistical analysis of the results shown in Figure 1 and 2, and a two-way ANOVA followed by Bonferroni analysis was performed for statistical analysis of the results shown in Figure 7. Differences with a p value<0.05 were considered statistically significant.

### Electrophoretic mobility shift assay (EMSA)

200 ng of annealed synthetic oligonucleotide probes were end-labeled with 6000 Ci/mmol [γ-^32^P]ATP (Perkin Elmer, Bosten, USA) by 10 U of T4 polynucleotide kinase (New England Biolabs) in a final reaction volume of 10 µl for 30 min at 37°C and purified from a 15% non-denaturing polyacrylamide gel. Approximately 0.2 ng (10–15000 cpm) of the probe was incubated together with either 25 ng of full length GST-tagged FOXO3 protein (Abnova, Taipei, Taiwan) or 2–4 µg of nuclear cell extracts for 30 min at 25°C in a final volume of 20 µl binding buffer containing 10% glycerol, 12 mM HEPES, pH 7.9, 4 mM Tris-HCl, pH 7.9, 60 mM KCl, 1 mM dithiothreitol, 0.6 mg/ml bovine serum albumin, 0.5 µg of poly(dI-dC) (Sigma) and competitor as indicated. For supershift assays, 2 µg of the corresponding antibody were added after 30 min and incubation was continued for 1 h at 4°C. Subsequently the binding reaction was separated on a 5.5–7% polyacrylamide gel in 1X TB 90 mM Tris, 90 mM boric acid).

### Chromatin Immunoprecipitation (ChIP)

ChIP assays were performed as described previously [47]. Briefly, 1×10^7^ of “444” cells were cross-linked with 1% formaldehyde for 10 min. The reaction was stopped by the addition of 0.125 mM glycine. Subsequently, cells were washed with phosphate-buffered saline (PBS) and lysed in ChIP-lysis buffer (5 mM HEPES pH 8, 85 mM KCl, 0.5% (v/v) Nonidet P-40, supplemented with 1 mM DTT, 5 mM NaF, 0.1 mM Na_3_VO_4_ and “Complete protease inhibitors” (Roche). Lysates were centrifuged to remove debris and resuspended in ChIP-buffer (20 mM Tris pH 8, 1.2 mM EDTA, 200 mM NaCl, 0.01% SDS, 1.1% Triton X-100) supplemented with 1 mM DTT, 5 mM NaF, 0.1 mM Na_3_VO_4_ and “Complete protease inhibitors”(Roche). Then chromatin was sonicated to shear DNA to an average size of 500 bp, insoluble debris was removed by centrifugation and lysates (equivalent to 25 µg of DNA) were precleared adding 40 µl of protein A/protein G-agarose mixture (Roche) blocked with 40 µg of sheared salmon sperm DNA (Eppendorf) for 2 h at 4°C in a total volume of 500 µl ChIP-dilution buffer (16.7 mM Tris pH 8, 167 mM NaCl, 1.2 mM EDTA, 1.1% (v/v) Triton X-100 and 0.01% (v/v) SDS). After centrifugation, supernatants were incubated with 10 µg of the corresponding antibody for 14 h and protein-DNA complexes were precipitated for 3 h with 40 µl of protein A/protein G-agarose mixture. After extensive washing, the immunocomplexes were eluted in 250 µl of 0.1 M NaHCO_3_ containing 1% (v/v) SDS and cross-links were reversed by adding NaCl to a final concentration of 200 mM and vigorous mixing at 65°C for 14 h. After protein digestion with proteinase K (New England Biolabs), DNA was recovered by phenol-chloroform extraction and ethanol precipitation and analyzed by semiquantitative PCR using the forward primer (5′-GTGACCTACGACCCCCTTC-3′) and the reverse primer (5′-GCTGACGATTGGAGCGTTTG-3′) to amplify the LKB1 promoter region. Primers used for amplification of the LKB1 coding region encompassing Exon 4–5 were: forward (5′-TCAGCTGATTGACGGCCTGGA-3′) and reverse (5′-CCAGCCGACCAGATGTCCAC-3′).

### RNA Interference

Small interfering RNA (siRNA) targeting NF-YA mRNA [48] (sense strand, 5′-GUCCAGACCCUCCAGGUAG-dTdT-3′; antisense strand, 5′- CUACCUGGAGGGUCUGGAC-dTdT-3′) or mRNAs of all FOXO family members [49] (sense strand, 5′-AGGAUAAGGGCGACAGCAA-dTdT-3′; antisense strand, 5′- UUGCUGUCGCCCUUAUCCU-dTdT-3′) and non-targeting control [50] (DNA target sequence, 5′-AACAGTCGCGTTTGCGACTGG-3′) were purchased from Qiagen. Cells were transfected using the HiPerfect reagent (Qiagen) according to the manufacturer's instruction. For transfection-complex formation, 0.4 nmol of siRNA were mixed with 20 µl HiPerfect in 100 µl of serum free DMEM. After 10 min of incubation, C33a and “444” cells were seeded at a density of 5×10^5^/6 cm plate, IMR-90 at a density of 7.5×10^5^/6 cm plate in 4 ml of normal growth medium and 100 µl of the transfection-complex were added dropwise to the cell-suspension, leading to a final concentration of 100 nM siRNA in the culture medium. 48 h after transfection, cells were splitted and an identical second transfection was performed. 96 h after the first transfection, cells were lysed as described previously [30] and cytoplasmic RNA was isolated using the RNeasy Kit (Qiagen). cDNA was obtained from reverse transcription of 1 µg of total RNA using 10 ng/µl of p(dN)_6_ random primers (Roche) and SuperScript II reverse transcriptase (Invitrogen) in a final volume of 20 µl according to the manufacturer's instructions. 1 µl of cDNA was subsequently used for PCRs using Platinum Taq DNA polymerase (Invitrogen). All PCRs were monitored within the linear range, which has been determined for each reaction individually. PCR condidions for each reaction, including primers, annealing temperature, amplicon size and number of cycles, are listed in Table S1.

For Western blot analysis, total protein content was determined according to Bradford [51]. Cell lysates were separated by SDS-polyacrylamide gel electrophoresis (5 µg total protein per lane) on a 10% separating gel and transferred onto a PVDF-membrane (Millipore) using a TE 77 semi-dry transfer unit (Amersham Bioscience). Membranes were then blocked overnight at 4°C, using 5% of milk powder in TBST (0.15 M NaCl, 10 mM Tris, 0.05% (v/v) Tween 20, pH 8.0). Proteins of interest were visualized using the antibodies described above and the enhanced chemoluminescence substrate Plus-ECL (PerkinElmer).

## Supporting Information

Figure S1 **Deletion and substitution-mutant analysis of the LKB1 promoter.** (**A**) Comparison of luciferase activity of transiently transfected C33a cells with LKB1 promoter 20 bp deletion constructs (right) and predicted *cis*-regulatory elements (left). The positions of the potential CCAAT boxes I–III as well as the forkhead box are indicated. Luciferase activity of all deletion constructs (relative light units normalized against renilla luciferase activity) is expressed as the percentage of the signal obtained with the plasmid containing the LKB1 promoter region downstream of nucleotide −345 (LKB1 Pro III). (**B**) Luciferase activity of transiently transfected C33a cells with LKB1 promoter 10 bp substitution mutants reveals a critical role of four *cis*-acting elements regulating LKB1 transcription (dark grey bars). Activity of substitution mutants (relative light units normalized against renilla luciferase activity) is expressed as the percentage of the signal obtained with the plasmid containing the LKB1 wild-type promoter (LKB1 Pro II, −549 to +727). Each bar represents the means ± standard deviation of three independent experiments made in quadruplicate.
(TIF)

Table S1 **Primer list.** Sequences of primers used for PCR-amplification of LKB1 promoter fragments, full length open reading frames (ORF) of indicated transcription factors, site-directed mutagenesis of FOXO3 and FOXO4 ORFs and cDNA of indicated mRNAs after reverse transcription (RT-PCR). For RT-PCR experiments the annealing temperature, the amplicon size as well as the number of conducted cycles is indicated.
(DOC)
