# Supplementary figures and images for: Gene Expression of the Tumour Suppressor LKB1 Is Mediated by Sp1, NF-Y and FOXO Transcription Factors
Source: PLoS One. 2012 Mar 6;7(3):e32590. doi: 10.1371/journal.pone.0032590 (PMC3295762; doi:10.1371/journal.pone.0032590)

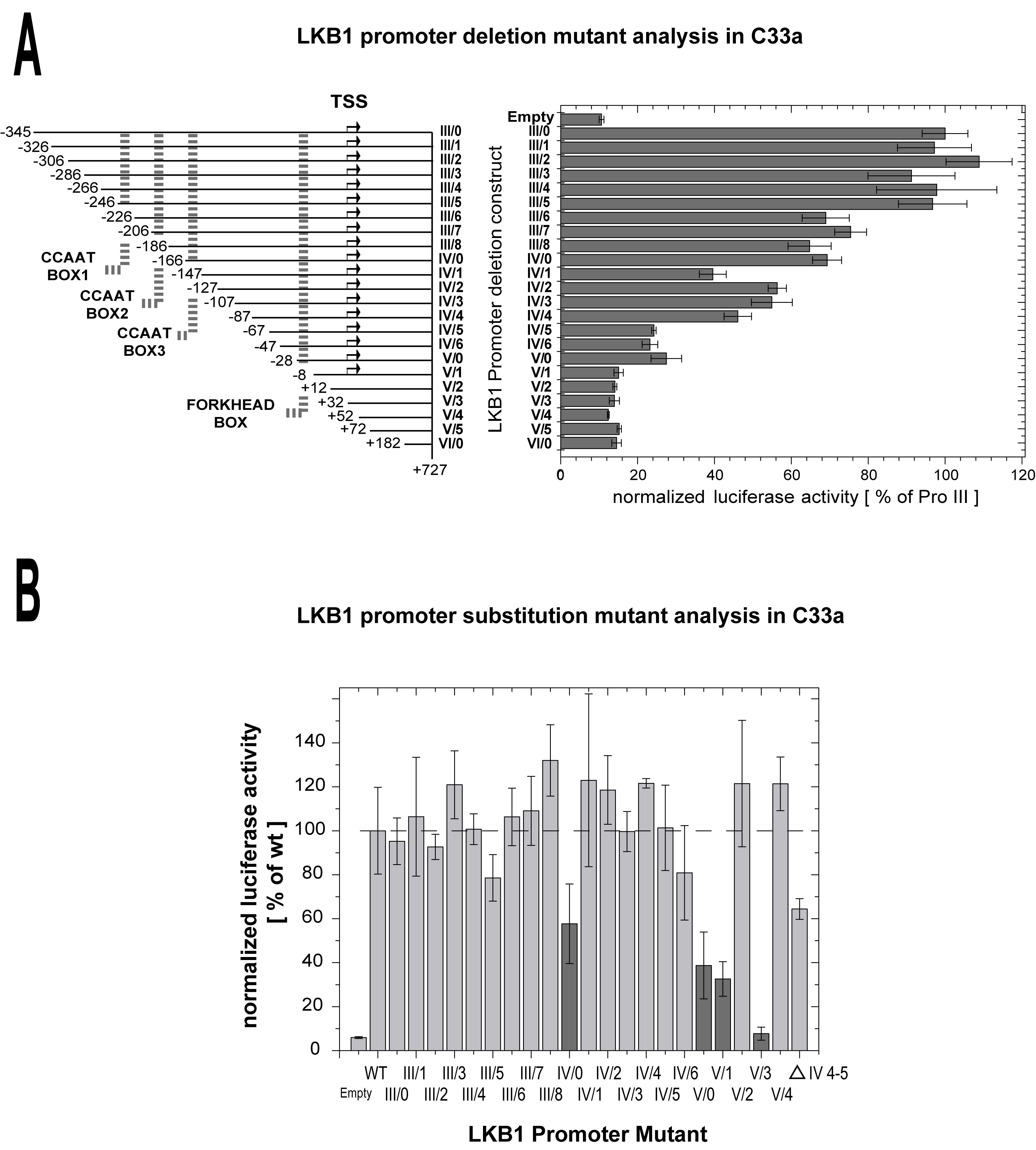

Supplement: Figure S1 — Deletion and substitution-mutant analysis of the LKB1 promoter. (A) Comparison of luciferase activity of transiently transfected C33a cells with LKB1 promoter 20 bp deletion constructs (right) and predicted cis-regulatory elements (left). The positions of the potential CCAAT boxes I–III as well as the forkhead box are indicated. Luciferase activity of all deletion constructs (relative light units normalized against renilla luciferase activity) is expressed as the percentage of the signal obtained with the plasmid containing the LKB1 promoter region downstream of nucleotide −345 (LKB1 Pro III). (B) Luciferase activity of transiently transfected C33a cells with LKB1 promoter 10 bp substitution mutants reveals a critical role of four cis-acting elements regulating LKB1 transcription (dark grey bars). Activity of substitution mutants (relative light units normalized against renilla luciferase activity) is expressed as the percentage of the signal obtained with the plasmid containing the LKB1 wild-type promoter (LKB1 Pro II, −549 to +727). Each bar represents the means ± standard deviation of three independent experiments made in quadruplicate. (TIF) [file pone.0032590.s001.tif]
