# Supplementary material for: Gene Expression of the Tumour Suppressor LKB1 Is Mediated by Sp1, NF-Y and FOXO Transcription Factors
Source: PLoS One. 2012 Mar 6;7(3):e32590. doi: 10.1371/journal.pone.0032590 (PMC3295762; doi:10.1371/journal.pone.0032590)
Supplement: Table S1 — Primer list. Sequences of primers used for PCR-amplification of LKB1 promoter fragments, full length open reading frames (ORF) of indicated transcription factors, site-directed mutagenesis of FOXO3 and FOXO4 ORFs and cDNA of indicated mRNAs after reverse transcription (RT-PCR). For RT-PCR experiments the annealing temperature, the amplicon size as well as the number of conducted cycles is indicated. (DOC) [file pone.0032590.s002.doc]

**Table S1. Primer list.**

| **Primer name** | **Sequence** |
| --- | --- |
| **LKB1 promoter deletion mutants** | |
| Forward primer | |
| LKB1 Pro I | CACCCTGCCTAATGTCCCTA |
| LKB1 Pro II | GGTCCAAGAGTGGCTCAAAA |
| LKB1 Pro III/0 | GTGACCTACGACCCCCTTC |
| LKB1 Pro III/1 | CCGAGCCCCACCGAGGTCACAG |
| LKB1 Pro III/2 | AGCCGTGGCCTCGTCTCCCCATG |
| LKB1 Pro III/3 | ATGCCTGCTTCCCGCCCCCTG |
| LKB1 Pro III/4 | GCCCGTGACGGGCGTCTCCGAG |
| LKB1 Pro III/5 | AGGACCAATGAGCGCGCTGTATC |
| LKB1 Pro III/6 | ATCCACCCCTCGGGCGGGGCCAAGC |
| LKB1 Pro III/7 | CAAGCGCCGACCAATCGCCG |
| LKB1 Pro III/8 | CTCGGGCGCCCGGCCGGGTCCAAAC |
| LKB1 Pro IV/0 | CAAACGCTCCAATCGTCAG |
| LKB1 Pro IV/1 | CGGCGGCGGGGCGGGCAGAG |
| LKB1 Pro IV/2 | GGCCGGGGATGGCAGGTTCAACC |
| LKB1 Pro IV/3 | ACCAACGGGTGGGCACGTCGTC |
| LKB1 Pro IV/4 | TCCTCGCGAGGAGGCGTGCC |
| LKB1 Pro IV/5 | CTGCGGCCGGGCGTGCGGTGTC |
| LKB1 Pro IV/6 | TCCGCGGCGGCGCAGGGAG |
| LKB1 Pro V/0 | GGGGAGGGAGGTAAACAAGA |
| LKB1 Pro V/1 | TGGCGGCGGCGTGTCGGGCG |
| LKB1 Pro V/2 | CGGAAGGGGGAGGCGGCCCG |
| LKB1 Pro V/3 | GGGCGCCCGCGAGTGAGGCG |
| LKB1 Pro V/4 | CGGGGCGGCGAAGGGAGCGC |
| LKB1 Pro V/5 | GGGTGGCGGCACTTGCTGCC |
| LKB1 Pro VI | GGCACCTTCGGGAACC |
| LKB1 ProVII | CCCTGAGCGGAGCTGTT |
| Reverse Primer | |
| LKB1 Pro Del Reverse | GCCCACGGACAAGTATGAAC |
| **LKB1 promoter substitution mutants** | |
| Forward primer | |
| LKB1 Pro Mut Forward | AATTGAGCTCGGTCCAAGAGTGGCTCAAAAGAAGG |
| Reverse primer | |
| LKB1 Pro Mut III/0 | GATCGCTAGCGAGTAGGTCACGGGTGCCAACGG |
| LKB1 Pro Mut III/1 | GATCGCTAGCCATGGGGCTCGGGAAGGGGGTCG |
| LKB1 Pro Mut III/2 | GATCGCTAGCTCGGCCACGGCTGTGACCTCGGT |
| LKB1 Pro Mut III/3 | GATCGCTAGCTCAAGCAGGCATGGGGAGACGAG |
| LKB1 Pro Mut III/4 | GATCGCTAGCTACGTCACGGGCAGGGGGCGGGA |
| LKB1 Pro Mut III/5 | GATCGCTAGCTACATTGGTCCTCGGAGACGCCC |
| LKB1 Pro Mut III/6 | GATCGCTAGCTCAGGGGTGGATACAGCGCGCTC |
| LKB1 Pro Mut III/7 | GATCGCTAGCTCTCGGCGCTTGGCCCCGCCCGA |
| LKB1 Pro Mut III/8 | GATCGCTAGCTAGGCGCCCGAGCGGCGATTGGT |
| LKB1 Pro Mut IV/0 | GATCGCTAGCGTGAGCGTTTGGACCCGGCCGGG |
| LKB1 Pro Mut IV/1 | GATCGCTAGCTACCCGCCGCCGCTGACGATTGG |
| LKB1 Pro Mut IV/2 | GATCGCTAGCAGATCCCCGGCCCTCTGCCCGCC |
| LKB1 Pro Mut IV/3 | GATCGCTAGCTGACCCGTTGGTTGAACCTGCCA |
| LKB1 Pro Mut IV/4 | GATCGCTAGCGACTCGCGAGGACGACGTGCCCA |
| LKB1 Pro Mut IV/5 | GATCGCTAGCTACCGGCCGCAGGGCACGCCTCC |
| LKB1 Pro Mut IV/6 | GATCGCTAGCTACGCCGCGGACACCGCACGCCC |
| LKB1 Pro Mut V/0 | GATCGCTAGCCACTCCCTCCCCCTCCCTGCGCC |
| LKB1 Pro Mut V/1 | GATCGCTAGCCAGCCGCCGCCATCTTGTTTACC |
| LKB1 Pro Mut V/2 | GATCGCTAGCTACCCCCTTCCGCGCCCGACACG |
| LKB1 Pro Mut V/3 | GATCGCTAGCGAGCGGGCGCCCCGGGCCGCCTC |
| LKB1 Pro Mut V/4 | GATCGCTAGCGACGCCGCCCCGCGCCTCACTCG |
| **cDNA amplification for eukaryotic expression plasmids** | |
| NFYA full ORF FW | GCACGGAGTGTACCTCACAGC |
| NFYA full ORF RV | GGCTGACTTCACCAGTCAAGGT |
| NFYC full ORF FW | GGAAACGGTGCAAACGGCGT |
| NFYC full ORF RV | GTTGGGTGTCCTTGGCCTTGCC |
| FOXO3 full ORF FW | ATGGCAGAGGCACCGGCTTC |
| FOXO3 full ORF RV | TCAGCCTGGCACCCAGCTCT |
| FOXO4 full ORF FW | ATGGATCCGGGGAATGAGAA |
| FOXO4 full ORF RV | TCAGGGATCTGGCTCAAAGT |
| **Site directed mutagenesis of FOXO3 and FOXO 4 ORFs**  (mutations are underlined) | |
| FOXO3 T32A FW | GATCCTGT**G**CGTGGCCCCTGCAAAGGCC |
| FOXO3 T32A RV | GCGGACGGCTCTGGGGCTCGAACTCC |
| FOXO3 S253A FW | GCGGGCTGTC**G**CCATGGACAATAGCAAC |
| FOXO3 S253A RV | CGCCGGGGGGCTTTTCCGCTCTTCC |
| FOXO3 S315A FW | CGCACCAAT**G**CTAACGCCAGCACAGTCAG |
| FOXO3 S315A RV | TGAACGGAAGTCCGTCCACGCATCCAGC |
| FOXO4 T32A FW | GCTCCTGC**G**CCTGGCCCCTTCCCCG |
| FOXO4 T32A RV | GGGGACGGCTCTGGGGTTCGAAGTCGG |
| FOXO4 S197A FW | GGGCCGCC**G**CCATGGATAGCAGCAGC |
| FOXO4 S197A RV | GGCGGCGGGGGGCTTTGCCGCTCTTG |
| FOXO4 S262A FW | GAAGCAGT**G**CAAATGCCAGCAGTGTCAGCACC |
| FOXO4 S262A RV | GTGGACGGAAGGTGGTCCACATATCGGCTTC |

| RT-PCR | | | | |
| --- | --- | --- | --- | --- |
| **Primer name** | **Sequence** | Annealing  temperature | Amplicon  size | Conducted  Cycles  (within the linear range) |
| NF-YA FW | GTGCAGTTGCAGACTGAGGC | 59°C | 320 bp | 30 |
| NF-YA RV | GTCTGGGGCTGCTGGATGAT |
| NF-YB FW | GGTGCCATCAAGAGAAACGG | 57°C | 161 bp | 26 |
| NF-YB RV | AGCTGTGACTGCTCCACCAA |
| FOXO3 FW | GGGAAACCTGTCCTACGCGG | 59°C | 407 bp | 27 |
| FOXO3 RV | CAGGCCACTTGGAGAGCTGG |
| FOXO4 FW | GAGCCGATCCTGTTGCCCTC | 59°C | 507 bp | 30 |
| FOXO4 RV | GACAGGGCTCGTTGGAGTGG |
| GAPDH FW | GCCACATCGCTCAGACACCA | 59°C | 291 bp | 22 |
| GAPDH RV | CCAGCATCGCCCCACTTGAT |
| LKB1 FW | GAGCTGATGTCGGTGGGTAT | 55°C | 269 bp | 30 |
| LKB1 RV | CTTCACCTTGCCGTAAGAGC |
